# Managing a large incisional hernia in an obese and immunosuppressed patient: A case report

**DOI:** 10.1016/j.ijscr.2025.111029

**Published:** 2025-02-07

**Authors:** E.A.J. Alkemade, A.G. Baranski

**Affiliations:** a Leiden University Medical Center, Leiden, the Netherlands; b Abdominal Organ Transplant Centre, Department of Surgery, Leiden University Medical Center, Leiden, the Netherlands

**Keywords:** Kidney transplantation, Incisional hernia, Biologic mesh, Abdominal wall reconstruction, Immunosuppression, Case report

## Abstract

**Background:**

Large incisional hernias in high-risk patients, such as those undergoing immunosuppressive therapy, represent an extra surgical challenge due to elevated risks of infection and poor wound healing. This case report details the reconstruction of an abdominal wall defect in a high-risk patient using a high-cost yet robust and effective biologic mesh.

**Case presentation:**

A 38-year-old obese female with multiple comorbidities developed an LIH and an incarcerated left-sided inguinal hernia following a kidney transplantation. The surgical approach involved a two-layer mesh reconstruction, combining a biologic intraperitoneal mesh and an absorbable onlay mesh. To reduce the risk of infection and provide extra reinforcement, the hernia sac was preserved and sutured over the biologic mesh. Postoperative complications, including infection and seroma formation, were managed effectively with negative pressure wound therapy. The wound closed after seven months, with no recurrence observed during follow-up.

**Discussion:**

The biologic mesh, combined with the well-vascularized sac, demonstrated integration with vascularized tissue, minimizing infection risk, and providing natural reinforcement and enhanced healing. Advanced wound management, including negative pressure wound therapy, effectively resolved postoperative complications such as infection and seroma.

**Conclusion:**

This case demonstrates the use of a multilayer reconstruction approach that combines a biologic mesh and the reuse of the hernia sac, offering a viable option for managing complex hernias in high-risk, immunosuppressed patients. This technique minimizes infection risk and provides stable long-term outcomes, even in challenging clinical settings.

## Highlights

• Preserving the vascularized hernia sac promoted healing and reduced postoperative infection risk.
• Multilayer reconstruction with biologic mesh and hernia sac reuse provides an effective approach for complex hernias.
• Biologic mesh reduced infection rates and ensured lasting hernia repair in an immunosuppressed patient.

## Abbreviations

IH: Incisional hernias
LIH: Large incision hernias
NPWT: Negative pressure wound therapy
CT: Computed tomography

## 1 Introduction

Incisional hernias (IH) are a common complication after kidney transplantation, occurring in 1.8–18.0 % of the cases. Despite advancements in closure techniques, the incidence remains consistent. This suggests that components beyond surgical technique contribute to closure failure, including patient related, disease related and technical related factors [[1], [2], [3]]. Patient related risk factors for developing an IH are Diabetes Mellitus, obesity (BMI > 30) and the use of immunosuppressants, which are, however, essential after a transplantation [4]. Moreover, infection at the previous surgical site, as well as the choice of suture material and surgical technique, are contributors in the development of an IH [[5], [6], [7], [8], [9]]. There are various techniques for repairing an abdominal hernia. All techniques eventually share the same goal, to restore the displaced anatomy, reinforcement of the abdominal wall and preventing recurrence of the IH. The choice of surgical approach depends on several components, including the size of the IH, condition of the patient and the probability of complications. Among all IH, 15 % to 47 % can be classified as large incision hernias (LIH) (≥ 10 cm), according to the European Hernia Society (EHS) [10].

A study by Burger et al. (2004) showed that recurrence rates are significantly lower when using a mesh, which is now the standard approach for most hernia cases. Meshes can be broadly categorized into two main types: biologic and synthetic. The choice of mesh and location depends on the complexity of the hernia and the patient related factors [[11], [12], [13], [14]]. Biologic meshes are significantly more expensive than synthetic meshes, making their use not feasible for all patients. However, certain high-risk patients may get greater benefits from biologic meshes. Strattice™ is a biologic mesh which is used during the repair of the LIH for our patient. This mesh is derived from porcine skin and undergoes processing to reduce the likelihood of xenogeneic rejection. The preparation process involves cellular removal and a reduction in the presence of α-Gal antigens [15,16]. The mesh offers several advantages, including the facilitation of rapid revascularization, collagen remodeling, and the migration of white blood cells. These features make it particularly suitable for contaminated or compromised wounds, as it integrates effectively with surrounding tissue and poses a lower risk of infection compared to synthetic meshes [17,19,20].

This case report highlights the challenges and complexities associated with managing an LIH in a high-risk patient. Immunosuppression, obesity, and prior surgical site infections significantly complicate hernia management, therefore demanding creative solutions. The treatment approach demonstrates the use of a high-cost yet robust and effective biologic mesh in combination with the reuse of a well-vascularized hernia sac. By presenting this case, we aim to provide insights into advanced techniques for LIH repair and their role in improving outcomes for high-risk patients with complex abdominal wall defects. This case report is reported in line with the SCARE criteria [19].

## 2 Case report

A 38-year-old obese female (BMI = 45) with kidney failure underwent a kidney transplantation in early March 2016, with the graft placed in the right lower abdomen. Medical history included Diabetes Mellitus Type I, with a surgical history of an amputation of the left lower leg (1999) and a hysterectomy (2005). While the kidney transplantation was technically successful, the postoperative course was complicated.

Within two weeks, the patient developed an abdominal wound dehiscence, requiring surgical intervention. Two days later, blood was detected in the surgical drain, necessitating a second operation. On April 12th, the patient underwent a third surgery for the fascial dehiscence, secondary to an incarcerated small bowel obstruction caused by a left-sided inguinal hernia. Intraoperatively, no abnormalities or active bleeding were observed, and three drains were placed. Additionally, the patient developed a surgical site infection, which, in combination with poor wound healing, resulted in further weakening of the abdominal wall, along with necrosis of the abdominal wall tissue. Surgical debridement was performed to remove the necrotic tissue; however, due to the extent of the damage, definitive wound closure was not feasible. At the time, the solution involved a temporary closure of the wound using only the subcutaneous tissue and skin layers.

Further complicating the situation, the patient experienced acute humoral rejection with acute tubular necrosis. Treatment included plasmapheresis and solumedrol, which delayed postoperative recovery. After stabilization, the patient was discharged home with a LIH, resulting from the previous complications.

**Fig. 1:**
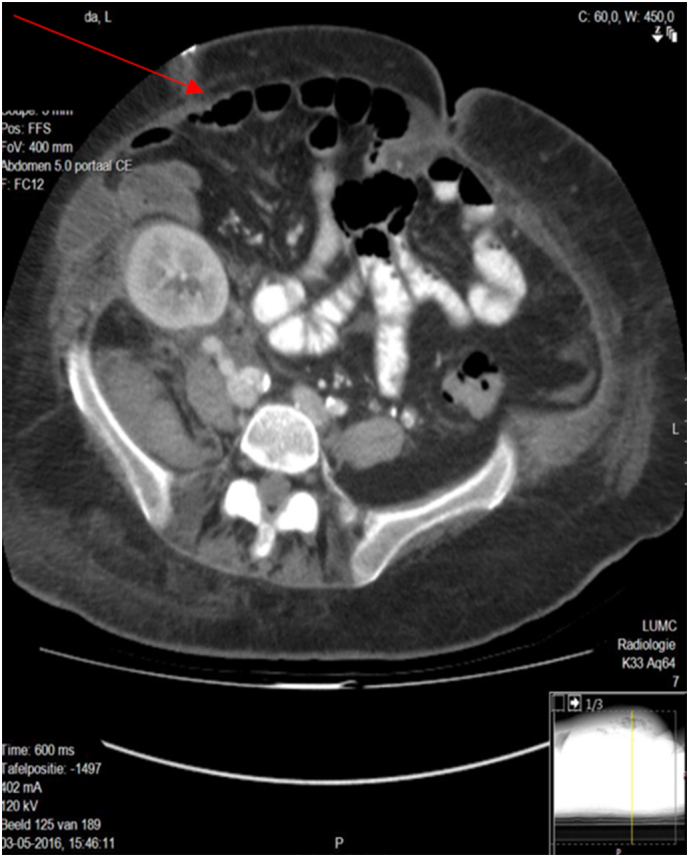
A transverse section of an abdominal Computed tomography (CT) showing the LIH (red arrow).

**Fig. 2:**
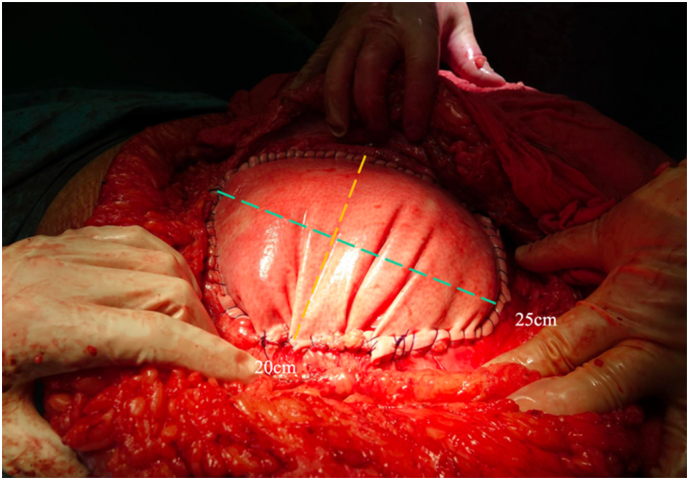
In the missing part of the abdominal wall biological mesh (Strattice™) was placed intraperitoneally and sutured.

In December 2016, a laparotomy was performed through the previous kidney transplantation incision to repair the LIH. Prior to the repair, a CT scan was performed, as illustrated in Fig. 1. Due to the absence of fascia in the abdominal wall, traditional reconstructive techniques, such as CST, were not feasible. Instead, a biological mesh of 20 × 25 cm (Strattice™) was chosen for bridging the fascia, as demonstrated in Fig. 2. The mesh was placed intraperitoneally, directly covering the large defect in the abdominal wall. The well-vascularized hernia sac was preserved and sewn to the anterior surface of the abdominal wall over the biological mesh (Strattice™) providing natural reinforcement and ensuring complete coverage. A Jackson-Pratt drain was placed between the hernia sac and the Strattice™ mesh to prevent fluid collection.

**Fig. 3:**
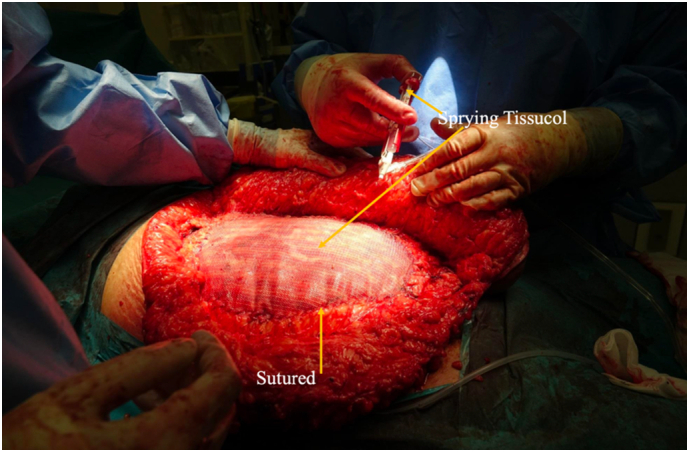
The reinforcement of the abdominal wall by adding onlay mesh (Phasix-Bard™). Edges of the mesh were sutured to the abdominal wall (yellow arrow) and the center of the mesh was connected via Tissucol (yellow arrow). (For interpretation of the references to colour in this figure legend, the reader is referred to the web version of this article.)

An absorbable mesh (Phasix-Bard™) was added as a secondary layer to further strengthen the reconstruction. It offers temporary mechanical support and promotes collagen formation as it degrades. The mesh was sutured at the edges to the abdominal wall and secured centrally with Tissucol (fibrin glue), minimizing tissue tension and promoting uniform attachment, as shown in Fig. 3. White sponges were placed to reduce infection risk and facilitate postoperative negative pressure wound therapy (NPWT).

Despite the comprehensive repair, there were postoperative complications. Within three weeks, the patient developed a wound infection managed with open wound care and continued NPWT. Seroma formed between the biological mesh and hernia sac, which became infected with Morganella, necessitating drainage and a course of antibiotics. Nevertheless, the patient's condition gradually improved. By two months postoperatively, the seroma had largely resolved, and skin edges were healing under continued NPWT. After seven months, the wound was fully closed and healed. At the end of the recovery period, the abdominal wall remained stable, the patient had good kidney function, and no recurrence of hernias was observed.

## 3 Discussion

Our high-risk patient underwent a kidney transplantation in March 2016, followed by a complicated recovery including a LIH and an incarcerated left-sided inguinal hernia, which resulted in a small bowel obstruction. With a BMI of 45, the patient was classified as severely obese, which increases intra-abdominal pressure. Additionally, she was on immunosuppressive therapy due to her kidney transplantation. In April, she developed symptoms of acute humoral rejection and was treated with plasmapheresis and solumedrol. This, combined with her diabetes mellitus, contributed to impaired wound healing [[1], [2], [3],^9^,^20^]. Therefore, the treatment plan for the patient required a creative approach, incorporating advanced materials and surgical techniques.

The biologic mesh (Strattice™) was positioned intraperitoneally to repair the defect and compensate for the absence of fascia. The choice of mesh was based on the bacterial index, with biologic materials being particularly suited for contaminated or compromised wounds due to their ability to integrate with vascularized tissue and lower infection risk compared to synthetic meshes [17,18]. This approach is supported by findings from Itani et al. (2012) who demonstrated lower infection rates with biologic meshes in contaminated surgical environments [21]. Such evidence reinforces the suitability of biologic mesh in managing complex cases like this one, where synthetic options would pose higher risks of failure or additional surgical interventions.

Synthetic meshes lack a vascular network, which makes them more susceptible to persistent infections. If infected, the wound often requires temporary closure and further surgical intervention after the infection resolves, delaying definitive repair. In contrast, biologic meshes can remain in situ during infection, enabling continued healing and reducing the need for additional procedures [[21], [22], [23]]. Therefore, the usage of the Strattice™ mesh was a suitable choice since our patient uses immunosuppressants, which increase the risk of postoperative infection.

Another aspect of this reconstruction was the reuse of the hernia sac as a natural reinforcement. Rather than being removed, the hernia sac was used to completely cover the biologic mesh. This approach takes advantage of the sac's natural vascularization, which is critical for tissue healing and minimizing the risk of infections or material displacement. The strategy of preserving a well-vascularized hernia sac is supported by findings from a systematic review by Chaouch et al. (2023), which suggests that sac reduction techniques can enhance vascularization and promote tissue healing [24,25].

An absorbable mesh (Phasix-Bard™) was placed in an onlay position over the well vascularized hernia sac providing an extra layer of reinforcement and serving as temporary support during the regeneration of connective tissue. The onlay mesh provides additional protection without introducing tension to the weakened fascial edges [4]. Tissucol was used combined with sutures for securing the Phasix-Bard™ mesh. In complex cases like contaminated or high-risk incisional hernia repairs, fibrin glue offers the advantage of reducing tissue trauma during fixation. A study by Petter-Puchner et al. (2008) specifically assessed the use of fibrin glue in the repair of IH with condensed synthetic meshes and demonstrated enhanced tissue integration and reduced adhesion formation, contributing to improved postoperative outcomes [26,27].

This case highlights the reconstructive techniques in managing complex LIH in high-risk patients. Thanks to the implantation of a biologic mesh capable of combating infection combined with the well-vascularized hernia sac, it was possible to successfully drain the infected seroma and deliver targeted antibiotics directly to the site of infection, even in an immunosuppressed patient. As a result, the patient achieved complete recovery, with a well-functioning kidney, a durable hernia repair, and no recurrence of infection. This outcome illustrates how the careful selection of advanced materials and surgical techniques can effectively address the challenges posed by complex cases, offering valuable insights for similar clinical scenarios.

## CRediT authorship contribution statement

All authors contributed to the conception and design of the research and writing of the manuscript.

## Consent

Informed consent was obtained.

## Ethical approval

A case report is not considered scientific research. Therefore, it does not require approval or agreement from the Medical Ethics Review Committee (METC).

Reference: medisch-etische toetsingcommissie Leiden-Den Haag-Delft.

## Guarantor

The corresponding author accepts full responsibility for the work.

## Research registration number

Not applicable. This research was not required to be registered in any research registry.

## Sources of funding

No sources of funding were used for this research.

## Declaration of competing interest

No conflicts of interest to declare.
